# Information Systems for Patient Follow-Up and Chronic Management of HIV and Tuberculosis: A Life-Saving Technology in Resource-Poor Areas

**DOI:** 10.2196/jmir.9.4.e29

**Published:** 2007-10-22

**Authors:** Hamish SF Fraser, Christian Allen, Christopher Bailey, Gerry Douglas, Sonya Shin, Joaquin Blaya

**Affiliations:** ^6^Harvard Medical School-MIT Division of Health Sciences & TechnologyCambridgeMAUSA; ^5^Baobab Health PartnershipLilongweMalawi; ^4^World Health OrganizationGenevaSwitzerland; ^3^Partners In Health RwandaRwinkwavuRwanda; ^2^Partners In HealthBostonMAUSA; ^1^Division of Social Medicine & Health InequalitiesBrigham & Women’s HospitalBostonMAUSA

**Keywords:** Anti-retroviral therapy, developing countries, information systems, patient follow-up, HIV/AIDS, Tuberculosis

## Abstract

**Background:**

The scale-up of treatment for HIV and multidrug-resistant tuberculosis (MDR-TB) in developing countries requires a long-term relationship with the patient, accurate and accessible records of each patient’s history, and methods to track his/her progress. Recent studies have shown up to 24% loss to follow-up of HIV patients in Africa during treatment and many patients not being started on treatment at all. Some programs for prevention of maternal–child transmission have more than 80% loss to follow-up of babies born to HIV-positive mothers. These patients are at great risk of dying or developing drug resistance if their antiretroviral therapy is interrupted. Similar problems have been found in the scale-up of MDR-TB treatment.

**Objectives:**

The aim of the study was to assess the role of medical information systems in tracking patients with HIV or MDR-TB, ensuring they are promptly started on high quality care, and reducing loss to follow-up.

**Methods:**

A literature search was conducted starting from a previous review and using Medline and Google Scholar. Due to the nature of this work and the relative lack of published articles to date, the authors also relied on personal knowledge and experience of systems in use and their own assessments of systems.

**Results:**

Functionality for tracking patients and detecting those lost to follow-up is described in six HIV and MDR-TB treatment projects in Africa and Latin America. Preliminary data show benefits in tracking patients who have not been prescribed appropriate drugs, those who fail to return for follow-up, and those who do not have medications picked up for them by health care workers. There were also benefits seen in providing access to key laboratory data and in using this data to improve the timeliness and quality of care. Follow-up was typically achieved by a combination of reports from information systems along with teams of community health care workers. New technologies such as low-cost satellite Internet access, personal digital assistants, and cell phones are helping to expand the reach of these systems.

**Conclusions:**

Effective information systems in developing countries are a recent innovation but will need to play an increasing role in supporting and monitoring HIV and MDR-TB projects as they scale up from thousands to hundreds of thousands of patients. A particular focus should be placed on tracking patients from initial diagnosis to initiation of effective treatment and then monitoring them for treatment breaks or loss to follow-up. More quantitative evaluations need to be performed on the impact of electronic information systems on tracking patients.

## Introduction

The scale-up of HIV treatment in developing countries, and the parallel creation of large-scale treatment programs for multidrug-resistant tuberculosis (MDR-TB), requires the creation of systems for chronic disease management in places where short-term care, or no care at all, is the tradition. Managing chronic diseases requires a long-term relationship with the patient and places additional demands on the health system, in particular the need to maintain a patient’s history and monitor his/her progress. One strategy to document chronic care is to give patients a small notebook or card, “a health passport,” for their records. As long as it is not lost or damaged (an important risk), this can solve the first problem—having a history of the patient’s care. The weakness is that it does not support the monitoring and surveillance of patient care. It puts the burden on the patient to take his/her treatment and to return for follow-up visits. Not only must the patient remember the date, find and pay for transport, and arrange child care, but the patient must also understand the importance of the treatment and the treatment plan. Particular at-risk groups are patients with HIV, partially treated tuberculosis (TB), or other chronic diseases who are asymptomatic and may not have the capacity to fulfill these requirements. Over 20% of patients on antiretroviral (ARV) treatment have missed appointments [1] or been lost to follow-up [2] in 1 year in some major HIV projects in Africa, with one study reporting 59% loss to follow-up over 4 years [3]. Many prevention of mother to child transmission (PMTCT) programs report very high loss to follow-up, with losses of more than 80% recorded in South Africa [4].

Lawn et al [5] identify three stages of the HIV treatment process that must be monitored for losses to follow-up or deaths: (1) patients with known HIV needing assessment and decisions on treatment, (2) patients in the first 4 months of ARV treatment, and (3) patients on long-term ARV therapy. Stages 2 and 3 have received the most attention to date, but stage 1 is also a major point of failure, with 46% of deaths in that study occurring prior to the start of treatment [5]. An additional group to consider is patients at high risk of contacting HIV, such as sexual contacts of index cases. The challenge of patient tracking and long-term follow-up is certain to grow as we move toward universal HIV testing and treatment in high-burden countries [6].

A solution to these challenges is an information system to track all patients with the disease, keep records of critical data such as laboratory tests and medication, and provide continual updates about treatment status. Given the barriers that can prevent a patient from returning for follow-up in resource-poor environments [7-9] and the frequent need for migration due to poverty and social disruption, the responsibility must be placed on the health system to find missing patients and provide treatment. Failure to do so for HIV and TB is often a life-threatening situation. An essential component of the information system is a master patient list, regularly updated and used to find missing or failing patients. This list should include a system for unique patient ID numbers. Our experience shows that if the project has more than one treatment site and/or more than a few hundred patients, this master patient list must be computerized and be part of an electronic medical record (EMR) system.

The objective of this review is to assess the role of medical information systems in tracking patients with HIV or MDR-TB, in ensuring they are promptly started on high quality care, and in reducing loss to follow-up.

### Information Challenges in Providing HIV Treatment

A typical patient diagnosed with HIV in resource-poor areas has a median survival of 5-10 years if untreated [10,11]. During this entire period, the patient is likely to infect others. Even diagnosing such patients is challenging, as a 2007 World Health Organization (WHO) report states: “Surveys in sub-Saharan Africa have shown that a median of just 12% of men and 10% of women had been tested for HIV and received the results” [12]. Until recently, providing access to treatment for known HIV patients was the greatest challenge. Now, as the scale of treatment programs increases, building on the WHO’s 3 by 5 Initiative [13], so does the challenge of identifying patients, placing them on appropriate treatment, and monitoring them. There are many critical steps that lead to successful long-term HIV treatment, as shown in Textbox 1.

**Critical steps in successful HIV treatment**

1. Outreach and screening

2. HIV testing

3. Patient assessment and/or WHO staging where CD4 count is not available

4. CD4 count

5. Prescription of medication by physician

6. Dispensing of medication from pharmacy

7. Receipt of medication by health institution, patient, or health care worker

8. Ensuring compliance with treatment through directly observed therapy, if possible

9. Following up the patient for complications and adverse events

At each step, the patient may be lost from the treatment process unless there are good systems in place to track his/her status. This process is complicated by several factors:

- The treatment process is spread over a wide geographic area. A patient may be tested and treated at different health institutions either because he/she moves between clinics or is referred to the ARV clinic.
- Laboratories may be far from the clinic, especially for CD4 counts.
- General infrastructure and systems are usually poor, resulting in a high risk that samples or test results may be lost.
- Staff are likely to be overworked and thus lack time to check for missing patients each month.
- It is difficult to contact patients because of poor specificity of the patient’s physical address due to absence of street names and house numbers.

### Challenges for Effective Prevention of Mother to Child Transmission Programs

A recent article reviewed the need to improve the organization of PMTCT programs to reduce the high risk of infants being born without HIV prophylaxis [1]. According to Reithinger et al, “the few studies that have attempted to evaluate such [PMTCT] programmes show the logistical, managerial, and technical challenges in delivering effective preventive services.” The authors also note that patients are progressively lost to the care process as they move along the care pathway, “thus, 12 months after delivery, only a fraction (19% in one study in Malawi) of HIV positive mothers who received antiretroviral drugs will attend health services to have their infant tested for HIV. Clearly, this may have lethal consequences for those children who become HIV positive” [1]. As the authors stress, a major reason for these problems is a lack of good information systems (paper or electronic) to track patients at risk. “For example, data on the number of pregnant women who tested HIV positive, received prophylaxis, and then delivered children who also received prophylaxis are not readily available. To obtain linked data, patient registers would need to be thoroughly cross-checked, which would be time consuming and cumbersome without computerization and planning” [1].

### Information Challenges in Providing Multidrug Resistant Tuberculosis Treatment

Similar problems exist for patients with MDR-TB who must be identified, tested, and monitored while on an appropriate second-line drug regimen for over 2 years. Drug susceptibility testing (DST), which identifies the infecting organism’s drug resistance pattern, allows for tailored regimens that result in better outcomes [14,15]. However, communication of these results between central and local laboratories and clinical facilities can take several months [16], and, in some cases, results may never arrive (Hurtado R, personal communication, 2006). Further, ensuring that all patients identified with MDR-TB are on an appropriate regimen requires a master patient list as described above for HIV patients.

We have identified the important steps in ensuring that a patient who is failing first-line TB therapy (directly observed treatment, short course [DOTS]) is started on an effective second-line drug regimen, as shown in Textbox 2.

**Critical steps in successful MDR-TB treatment (example from Peru)**

1. Detection of DOTS failure or direct infection

3. DST performed

4. DST reviewed (primary TB doctor)

5. Decision to start second-line treatment (TB committee)

6. Effective regimen prescribed (pulmonologist)

7. Regimen dispensed from pharmacy

8. Regimen received at health center

9. Therapy directly observed and patient monitored for complications

As with HIV, almost all of these steps have a risk of failure: a sample may be lost or contaminated, results may not be reported back to the health center, or an effective second-line regimen may not be prescribed or dispensed to the health center. Failure to communicate results or follow up with patients has serious implications for the outcome of individuals who have usually failed at least one course of treatment for TB and may have seriously compromised lung function. In addition, it increases the risk of amplification of drug resistance by exposing patients to inadequate regimens for longer. Finally, by leaving patients infectious for longer, it puts other people at risk of contracting this dangerous disease.

## Methods

Due to the nature of this work and the relative lack of published articles to date, this review does not rely solely on a literature review. As there is a pressing need to solve the problems described here, we used three strategies to assess the availability and impact of information systems to support HIV and MDR-TB treatment in developing countries.

### Literature Review

An exhaustive literature search showed that as of August 2007, the systems described below are the only ones that have been the subject of publication, though not all in peer-reviewed journals or conference proceedings. This search used a review of EMRs in developing countries [17] as a start and was supplemented by a search of Medline and Google Scholar using combinations of the following terms: electronic medical record, electronic health record, electronic patient record, developing countries, third world, resource poor settings. Potentially relevant articles were retrieved and their reference lists reviewed for additional articles. Additionally, colleagues were consulted to identify further unpublished systems. We also reviewed articles on the outcome of HIV and MDR-TB treatment in developing countries for information on the use and success of electronic information systems. The search was restricted to articles in English.

### Personal Knowledge and Experience of Systems in Use

The authors have worked in a broad range of developing countries with HIV and MDR-TB programs and have experience in the design and deployment of such systems. Through contacts with organizations including the WHO, the US Centers for Disease Control and Prevention (CDC), and academic and nongovernmental organizations active in this area, they are familiar with the most widely used systems.

### Assessments of Systems Performed by the Authors

The authors have performed both formal and informal evaluations of the systems being used by their colleagues and organizations in the field.

## Results

### Projects Using Electronic Medical Information Systems to Reduce Loss to Follow-Up of HIV Patients

In Zambia, an HIV treatment program [18] evaluated 29998 HIV-positive patients for ARV treatment from 2004 to 2005. The program initially lost 4870 patients (16%) who did not return for the second visit, at least 44% of whom were considered eligible for immediate commencement of ART therapy. Of the subgroup of 16198 patients who started ARV therapy, 21% were late for follow-up in November 2005. Using custom reports from their medical information system, community health care workers were able to track down 32% of these patients and remind them to return to the clinic (27% were dead, and the rest could not be traced). Patients who were found and reminded were more than twice as likely to return as those that could not be traced. However, only 28% actually returned after one attempt to remind them, which suggests that detecting missing patients is necessary but not sufficient to ensure continuity of care.

In the Central Plateau of Haiti, Partners In Health/Zamni Lasante (PIH/ZL) runs nine hospitals that provide HIV treatment as well as general medical care. PIH/ZL has deployed a Web-based medical record system, the HIV-EMR, to all nine sites [19]. All positive HIV tests are logged in the HIV-EMR by laboratory staff, with 10800 HIV patients recorded as of June 2007. New HIV patients are seen by a doctor and have a CD4 count done. The result is entered into the HIV-EMR by the lab technician. An automated email is sent to medical staff if a patient has a result below 350 and there is no record of ARV therapy in the system [20]. A preliminary retrospective study examined whether early entry of CD4 counts into the HIV-EMR was associated with prompt ARV treatment. It showed that for patients with CD4 counts between 101 and 350, those who had a CD4 count entered within 14 days had an odds ratio of 3.2 (*P* = .008) for starting treatment early (defined as within 14 days) compared to those without early CD4 entry (K. Greenwood, PIH, unpublished report). High-risk patients with CD4 counts below 100 were almost all treated within 1 week. While this study was observational, the strong association between early entry of a CD4 count in the EMR and early treatment merits further investigation.

For patients commencing ARV treatment, an initial intake form and all follow-up forms are entered into the HIV-EMR by an onsite data clerk. The HIV-EMR also automatically creates monthly reports listing patients with missing CD4 counts or with low CD4 counts and no ARV regimens, along with other potential problems such as missing weights. One of the most effective tools is a monthly medication list automatically generated from the EMR that is used to track medications prescribed and those collected by community health care workers. This allows the team to learn about patient deaths, transfers, and other issues that are then updated in the HIV-EMR. We have also found patients who were lost to follow-up after an initial HIV test and CD4 count but before they started treatment. We are working to detect these missing patients more quickly using the master list of all positive HIV tests in the HIV-EMR and regular reports to the clinical team.

In rural Rwanda, PIH runs a group of six clinics in and around Rwinkwavu, and data on HIV patients are managed with a newer version of the HIV-EMR based on the OpenMRS architecture (HIV-EMR 2.0) [21,22]. We implemented the same workflow as in Haiti for HIV test results, CD4 counts, weights, and ARV regimens, and similar reports for clinicians. These include reports that highlight potential problems at each clinic visit. The medical staff report that this has reduced the time they spend looking for laboratory results, and they strongly request that reports are available before each clinic. In addition, patient follow-up visits are logged through the entry of follow-up forms, and patients who fail to return are highlighted in monthly reports. This allowed staff to rapidly identify a serious decline in follow-up among patients who had stopped receiving food supplementation early in 2007. New strategies were implemented within 3 weeks, and clinic attendance rapidly returned to its original level of over 90%. An alternative way we use the HIV-EMR 2.0 to determine if patients are adhering to their regimen is by tracking when community health care workers return to collect medications.

In Eldoret, Western Kenya, an EMR system was developed and deployed in 2002 to document primary care clinic visits [23]. A new version of this system, the AMPATH medical record system (AMRS), was implemented to support HIV treatment as part of the Academic Model for the Prevention and Treatment of HIV/AIDS (AMPATH) project [2]. The AMRS is used to track patients who missed clinic appointments, and an outreach team is sent to follow them up and ensure they received treatment. The team carried out a retrospective analysis of the reasons why patients failed to return for treatment and took measures to eliminate the major barriers identified. Strategies included adding evening/weekend clinics for the 24% of patients who could not leave work and supporting transport to the clinic for the 12% who lacked the bus fare. Another 8% were classed as “too sick to come” and likely at serious risk. In addition, the AMRS data have been linked to the records of HIV-positive pregnant women in the PMTCT clinic to ensure that they receive treatment in the HIV clinic.

In Malawi, the Baobab Health Partnership has developed an information system using an innovative touch screen interface [24]. This system, launched in 2001, has been used to issue nationally unique IDs to more than half a million patients across three urban sites. Upon registration, each patient is given a bar coded label showing his/her ID number, which is then affixed to the patient’s health passport. Baobab has also developed versions of this system to support both voluntary counseling and testing and ARV treatment. At the Lighthouse Clinic in Lilongwe, a monitoring and evaluation team uses data collected by the system to identify patients who have likely run out of ARV medication and to initiate follow-up. Identifying such patients is a nontrivial process that takes many factors into account, including the last appointment date, quantity of medication dispensed at last visit (ranging from 2 weeks to 3 months), and medication remaining from the previous visit. This type of process clearly benefits from computerization.

### Projects Using Electronic Medical Information Systems to Track MDR-TB Laboratory Results and Patients

PIH developed a Web-based medical record system, the PIH-EMR [25,26], to track patients with MDR-TB, store their treatment regimens and outcomes, and monitor their current status. A recent addition to this is the e-Chasqui module [27] that tracks the progress of a sample sent for a smear, culture, or DST and reports the data back to the relevant health personnel (see Textbox 2).

A baseline study showed that 10% of TB culture and DST results took over 60 days to arrive at the health center from the regional laboratory [16]. With the implementation of the e-Chasqui electronic laboratory reporting system [27], preliminary data from the prospective intervention study showed that no result took more than 17 days to be seen by health personnel. Additionally, contaminated samples are reported back to the health centers promptly so that another sample may be sent. The PIH-EMR has a record of all the regimen changes and links them to the pharmacy that dispenses the key second-line medications. We have implemented a feedback system from e-Chasqui to warn of delays and failures in the system, the effects of which are being studied in a clinical trial at present. We are also implementing a feature to alert health care providers when drug-resistant patients experience excessive delay in starting appropriate therapy or fail to receive appropriate medications based on their DST. A final stage will be to expand the system to include all patients receiving first-line TB treatment as well.

### Mobile Technologies

Cell phone networks are currently extending throughout most developing countries, even to remote rural areas. These are increasingly supporting data services, such as General Packet Radio Service (GPRS), allowing mobile Internet access. Several projects have used cell phones to assist in patient follow-up and provide access to medical data such as laboratory tests. For example, in Rwanda, PIH sends SMS messages warning of abnormal lab results to clinical staff. They have also been tested as a way to encourage patient compliance with TB treatment in South Africa [28] and to monitor medication side effects for treatment of sexually transmitted infections in Peru [29]. Voxiva Inc [30] has set up systems in Peru and Rwanda that allow staff to report data on patient care through a voice menu system to a Web-based reporting system. The Rwandan government uses this to facilitate national reporting of HIV outcomes. These technologies have real potential but are at an early stage in development and evaluation [31] and do not replace the need for a well-designed medical record system that links together data from multiple sources. There are also serious concerns about patient confidentiality when HIV data are transmitted by phone, which may be easier to address in a Web-based system. The best use of cell phones for patient tracking is likely to be as a tool to link patients, community health care workers, and clinics, extending the health network to the remotest communities and assisting in the location of missing patients. In those sites where the cell phone networks are not available, personal digital assistants may be carried by mobile staff and linked to a networked computer back at the main site. An example of this is a system PIH developed to collect bacteriology data from remote clinics and laboratories in Peru [32].

## Discussion

The examples presented here show that electronic information systems can be deployed in resource-poor areas to support HIV and MDR-TB treatment. Furthermore, they show that, when used correctly, such systems can help ensure patients are started and maintained on treatment.

One important question is whether electronic systems are superior to paper in these functions. Paper patient record systems in resource-poor settings have traditionally been successful when patient volume has been low enough to be effectively managed with limited human resources. In vertical programs such as DOTS treatment for TB, the demands of the paper record system have been simplified by the standardization of medication, laboratory tests, and reporting. Simple paper-based approaches clearly do not work well in their present form for HIV treatment, especially not for PMTCT [1]. While paper systems can likely be improved, there is little evidence to date that they alone can address all the problems identified here, particularly with large numbers of patients.

Information technology (IT) plays an important role in developed countries, but the role of such systems in developing countries is still evolving. It is essential to avoid expensive and difficult-to-maintain IT investments if their benefits are unclear. While studies evaluating the effects of IT on patient outcomes in developing counties are lacking to date, the projects and studies described here show that there are essential tasks that are very difficult to accomplish without IT systems. Determining who has missed follow-up appointments in a group of almost 11000 patients in nine clinics in rural Haiti is virtually impossible with paper records alone. At least nine patient lists (each with about 1000 entries, frequent corrections, and additions) would have to be brought together in one room and cross-checked. If one of the lists was lost, vital data would be gone and patient confidentiality put at risk. Compare this to pulling up a page in the HIV-EMR and running a custom report that shows patients at a site who are missing follow-up forms, CD4 counts, or other data. Corrections and additions to the records, while time consuming, are available to the whole team immediately, and transferred patients can be located and duplicate records detected with patient-matching algorithms. For example, when all records were entered into the HIV-EMR in Haiti, we found 150 fewer patients on treatment than indicated by the paper records.

All the systems described here use unique patient ID numbers to help prevent duplicate records or misidentification. Several systems include a check digit [33] that reduces the chance of data entry errors leading to the wrong record. This is supplemented in the Baobab system with bar coded ID cards, which have proven to be a low-cost and easy-to-use solution for over 500000 records.

### Additional Benefits of Electronic Medical Records

Once the investment has been made in entering and cleaning the data in an EMR, it is available to generate multiple report types. The AMRS system in Kenya reduced the time taken to prepare reports for the government from days to minutes [33]. These data also assist in forecasting requirements for supplies such as medications [33,34] and in conducting observational research. Allowing multiple staff, including lab technicians, pharmacists, HIV nurses, and social workers, to access the system helps to ensure consistency and detect errors. It can also improve buy-in by directly benefiting those collecting the data. In the case of laboratory data, the benefits of a Web-based system are obvious when supra-national laboratories are performing the tests for another country. Getting DST results from a South African laboratory for a new MDR-TB project in Lesotho is clearly easier and more reliable with an Internet-accessible system than by routine mail. While email will suffice in some cases, it lacks organization for large data volumes and typically lacks good tools for security and confidentiality. We have also found that more than 10% of DST requests in Peru are duplicated due in part to lack of communication in the health system. For example, of 238 DSTs ordered in hospitals in Lima, only 42% were available to the TB clinic prior to implementing the e-Chasqui system. This duplication not only wastes money but ties up valuable lab resources, potentially delaying other patients’ treatment.

Important features of information systems for patient follow-up include:

- A master patient list with tools to detect duplicate patient records
- Internet-based (if covering more than one site) to detect duplicates and transfers
- Linked to critical laboratory data, such as HIV status, CD4 count, or DST
- Secure, encrypted data [20] with specific controls on access for different staff
- Designed to collect, analyze, and display longitudinal patient data
- Incorporates alerts and warnings to identify missing or high-risk patients
- Consistent, off-site data backup
- Good interface for data entry and viewing, with tools for data management

Other features can be important in the development and deployment of medical record systems, including those in resource-poor areas.

- Coding medical data with standard codes allows the comparison of data and patient outcomes between sites. This includes the use of codes such as the International Classification of Diseases, Tenth Revision (ICD10) for diagnoses and International Nonproprietary Names (INN) for medications. The OpenMRS system uses a concept dictionary [21] to represent all data items about patients, including drugs, laboratory tests, and questions and answers on forms. This allows new items to be added by nonprogrammers. This dictionary is also mapped to standard coding systems. Similar designs are used in the Baobab system in Malawi and systems in Zambia. Such coding is important in creating decision support tools, for example, to warn of potential adverse events from medication [35]. Decision support rules are more reliable and easier to maintain if data are coded with standard systems.
- As life and death decisions may be made based on a medical record, it is important to know if any data items were changed and, if so, by whom and why. This helps to ensure high quality data and determine why data errors occur. In a system like OpenMRS [21], this is straightforward as each data item entered is a row in an “observations” table linked to the concept dictionary. If data need to be changed, the entry in the observation table is marked as “voided” with a reason, the name of the person who changed it, and the date. A new row is then entered with the changed value.

### Comparison with Prior Work

Studies in the United States and Israel have shown that integration of electronic laboratory-based reporting software decreases communication time and increases the completeness of reporting [36-39]. Studies have also shown that such systems can be used to warn doctors about important and urgent interventions [40-45]. The benefits of these alerts include (1) having more clinicians order an appropriate test for their patients [41,42,45], (2) a decrease in the time until an appropriate treatment was ordered for patients who had critical laboratory results [40,42,43], (3) improved patient outcomes after a specified time [43,44], and (4) fewer required follow-up visits [45]. We could not, however, find similar studies in resource-poor areas.

### Limitations

Although there are technically successful systems in resource-poor areas, as described here, one of the biggest challenges is data management—ensuring timely and accurate data entry. This is a long-term issue and has to be planned for with appropriate funds for training and staffing. The cost of setting up IT systems in resource-poor areas is clearly a barrier to many underfunded projects, particularly if only one application is supported. If the systems are well designed and flexible, the need for reporting to funders and governments can help to support the clinical benefits of patient tracking. There is also the need for long-term support and development of software systems and the resources this requires [17]. This is one of the benefits of collaborating with other organizations to build good information systems, as we have done with the OpenMRS cooperative [21].

A limitation of this review is the relatively small number of systems that have achieved widespread use and the lack of published evaluations, or even descriptions, of these systems. We have attempted to circumvent this problem by using personal experience of such systems in use. This has the risk of biasing the results to those systems known to the authors but should help to show the potential of such systems and encourage other researchers to explore this areas and report their work. Another limitation is the shortage of information on the costs of setting up and maintaining these systems, though one such study is being performed on the e-Chasqui system in Peru.

### Conclusions

To effectively treat HIV we must use medications and laboratory tests developed in the last two decades. Recent initiatives have made these items available in the poorest regions of the world, and pioneering treatment projects have shown that patients who are treated can have excellent outcomes [46-48]. Similar issues affect the treatment of MDR-TB, and since MDR-TB and HIV are frequently seen in the same patients, especially in southern Africa, they must be treated in a coordinated way [49,50]. The challenge now is to deliver treatment to vast numbers of patients and ensure that each receives excellent long-term care. Many efforts have been undertaken to meet this challenge, including, in particular, the internationally agreed treatment target of 778000 MDR-TB patients by 2015 [51], which is a huge scale-up from current numbers.

The evidence presented here shows that achieving such outcomes likely requires cutting-edge information systems carefully engineered for and tested in these challenging environments. The best medications and clinical expertise are of limited use if large numbers of patients are lost to the health system. It should be stressed that the poorest patients in the most remote areas are some of the most at risk of loss to follow-up [9] and therefore are the ones that most need effective information systems. Information systems can also result in other potential benefits, including tracking and preventing adverse events to medications and supporting less experienced health care workers in the follow-up of HIV patients.

Combining technology in the form of information systems for patient tracking with the local human capital of community health care workers who directly link to patients [52] should greatly improve treatment compliance, follow-up, and outcomes in these challenging areas. This approach should also reduce the emergence of dangerous drug resistance, which has become increasingly difficult and costly to treat, both with TB and, more recently, HIV. Finally, these approaches will also be of benefit to the management of other chronic diseases that are of growing importance worldwide, including diabetes, hypertension, and heart failure.

This review has highlighted the limited data available on the use of such information systems in resource-poor areas. There is an urgent need for further innovation in this area and for studies to evaluate the best ways of using information systems to strengthen clinical teams and patient care as part of the expansion of HIV and TB care worldwide.

## Abbreviations

AMPATH: Academic Model for the Prevention and Treatment of HIV/AIDS
AMRS: AMPATH medical record system
DOTS: directly observed treatment, short course
DST: drug susceptibility testing
EMR: electronic medical record
HIV: human immunodeficiency virus
IT: information technology
MDR-TB: multidrug-resistant tuberculosis
PIH: Partners In Health
PMTCT: prevention of mother to child transmission
TB: tuberculosis
WHO: World Health Organization
